# Position Estimation and Compensation Based on a Two-Step Extended Sliding-Mode Observer for a MSFESS

**DOI:** 10.3390/s18082467

**Published:** 2018-07-30

**Authors:** Shusheng Li, Yongling Fu, Ping Liu

**Affiliations:** 1 Beijing University of Aeronautics and Astronautics, Beijing 100191, China; lss123048@163.com; 2 PowerChina Road Bridge Group Company Limited, Beijing 100048, China; liuping8806@163.com

**Keywords:** magnetically suspended flywheel energy storage system, rotor position estimation, two-step extended sliding-mode observer, *H_∞_* optimization control

## Abstract

This paper aims to deal with the problem of rotor position estimation and compensation for a magnetically suspended flywheel energy storage system under the consideration of measurement noise and unknown disturbances. First, the flywheel system working principle and description are analyzed and, based on this, the mathematical model as well as the coordinates transformation are introduced. For the purpose of the state estimation, a two-step extended sliding-mode observer is considered to obtain the estimates of the rotor angular position. In this control strategy, a traditional sliding-mode observer is adopted as a first-step original state estimator. After that, the relationship between the angular position and the estimation error is established and a second-step observer is designed to obtain the estimation of the error. The estimated error is then used to compensate the real values of the rotor angular position generated by the first-step observer. To reject the influences of the measurement noise and unknown disturbances, the *H_∞_* optimization strategy is considered to determine the second-step observer structure. Finally, experimental results are presented to demonstrate the effectiveness of the proposed method. It is demonstrated that the proposed two-step observer method has a better estimation accuracy and control performance.

## 1. Introduction

As a novel physical energy storage technology, the magnetically suspended flywheel energy storage system (MSFESS) has the advantages of rapid charging-discharging process, high density and efficiency of energy and power, long life-period with small environment pollution and etc. [1,2,3]. Due to these priorities, the MSFESS has been widely used in the periods of rail traffic energy recovery, electric power magnitude regulation, uninterruptible power supply (UPS), electromagnetic launch and so on. Probably, the MSFESS contains two working modes: charging mode and discharging mode, which can be guaranteed with the active magnetic bearing (AMB), vacuum equipment, flywheel rotor and the motor/generator stator. In the charging mode, the flywheel rotor is driven by the motor stator for a high-speed rotation so that the electric energy can be stored in the form of kinetic energy. In the discharging mode, the motor stator is actuated by the flywheel rotor so that the kinetic energy can be transferred to the electric energy used by the load, in which the flywheel rotor has to decrease the speed gradually. Owing to the advantages of simple structure, small mass and volume, high efficiency and power factor and etc., the permanent magnet synchronous motor (PMSM) is the ideal selection as the driven device for the high-speed flywheel rotor to accomplish the charging-discharging control process [4,5].

In real practice, the vector control strategy is commonly used in PMSM control to improve the speed and current performance, in which the accurate rotor angular position is needed for coordinate transformation. The traditional method to obtain the rotor position is the sensor detection strategy such as resolver, encoder and hall device, which can be the simplest efficient measure by considering the real operation circumstance. But for magnetically suspended devices like MSFESS, twyer and compressor, the rotor is suspended in the seal house and there is a certain gap between the rotor and the body. In this case, a large displacement variation occurs in both axial and radial directions when the rotor begins to suspend or stays static. In addition, it is a common phenomenon that the rotor displacement can vary in real time, which requires a more difficult installation accuracy for the position sensor and a higher cost and reducer reliability. Therefore, the sensorless control strategy has become a leading issue in the PMSM control field [6,7,8,9].

In general, several studies pay attention to the sensorless control strategy of the PMSM: the first method relies on the magnetic path asymmetry of the permanent magnet motor. In this method, additional voltage and current signals are needed to produce a high-frequency excitation, in which the rotor position can be obtained by measuring the system response. This kind of method includes high-frequency signal injection, motor inductance detection and etc. [10,11,12,13]. However, the high-frequency signal excitation method is appropriate for the salient pole PMSM, which has a high-precision requirement for the physical and magnetic structure. The second one is the model estimation method such as the back electromotive force integration method, model reference self-adaption, sliding mode observer, extended Kalman filter and etc. [14,15,16]. The back electromotive force integration method has a wide application due to its simplicity and reliability, but this method relies on the motor stator voltage and current information to calculate the vector phase. When the current is small or zero, the rotor position cannot be obtained, resulting in a step failing out. The model reference self-adaption method contains an adjustment or reference model and a self-adaption scheme, which has a better capability of disturbance rejection, but this method depends on an accurate mathematical model and easily results in estimation error. The extended Kalman filter is an optimal full-order estimator, which can simultaneously reject the measurement noise and unknown disturbance, but this filter requires a complicated algorithm calculation and needs to be done with a powerful processor.

Among the mode-based estimation methods, the sliding-mode observer method has received more and more attention [17,18,19]. The observer designs the current error as the sliding-mode surface and based on this, the states can be converted to the real values by establishing an appropriate sliding-mode function. Due to the robustness regarding model parameters, the sliding-mode observer has been applied in many real experimental systems. However, a low-pass filter has to be used in this observer since there is a certain noise in the current information, resulting in an estimation phase delay and accuracy decline that need to be compensated for real time. To solve this problem, several modified observer strategies have been proposed to improve the estimation accuracy, such as extended sliding-mode observer and robust non-linear observer and etc., but these observers have not disposed the phase delay radically so that the estimation performance can be affected. In a previous study [20], a kind of phase-locked loop-based sliding-mode observer was used, which can improve the state estimation accuracy in place, but the control scheme is difficult to realize in practice. Above all, for the sensorless control strategy of the PMSM, the first physical estimation strategy can be fixable for the high-speed condition, and the estimation performance cannot be guaranteed, while the observer strategy has a certain phase delay and estimation error. In the whole speed areas, the sensorless control strategy is still a research emphasis, and there is difficulty for PMSM high-precision control.

Motivated by this, this paper proposes a two-step extended sliding-mode observer-based control strategy. The first-step observer adapts a traditional extended sliding-mode observer with a phase compensation to obtain the original estimated states, and the second-step establishes the error model and uses the *H_∞_* optimization strategy to reject the influences of unknown disturbances. To the authors’ knowledge, the *H_∞_* optimization control method has a priority and robustness against unknown disturbances [21,22,23]. Therefore, this paper aims to apply this method to the rotor position estimation and compensation for the MSFESS. This paper is organized as follows. Firstly, in Section 2, the system model and description are introduced. Section 3 gives the basic idea of this work, with the design of the sliding-mode observer proposed in Section 4. Experimental setup and results are presented in Section 5. Finally, Section 6 concludes this paper.

## 2. System Modeling and Description

**Figure 1:**
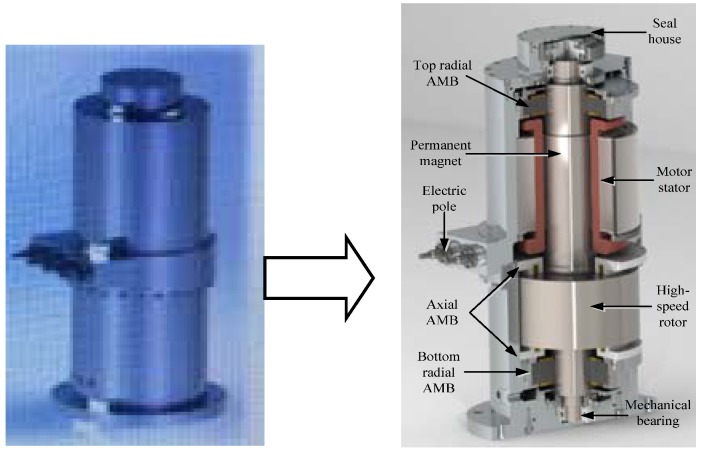
Schematic diagram of a magnetically suspended flywheel energy storage system (MSFESS).

As shown in Figure 1, the MEFESS is composed of the seal house, PMSM, AMB, electric pole mechanical bearing, etc. The PMSM is the crucial component for energy transmission in the form of electric and kinetic, which is divided into a motor stator and coaxial permanent magnet. The AMB possesses a 5-degree freedom in the axial and radial directions, which is able to maintain the high-speed rotor suspending in the vacuum body. The high-speed rotor is used as the kinetic energy storage component, and the mechanical bearing provides the strength protection once the AMB is running out. Outside of them, the seal house is reinforced to fix all of the components and thus provides a vacuum.

**Figure 2:**
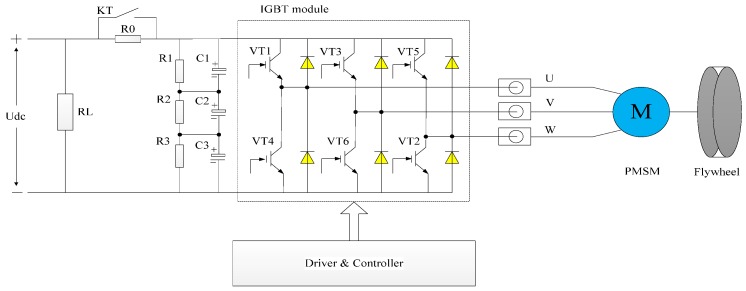
Minimum power scheme of permanent magnet synchronous motor (PMSM) driver. IGBT: insulated gate bipolar transistor.

Considering the problem of rotor strength during high-speed rotation, the PMSM adopts a mechanism of a surface-mounted permanent magnet with a high-intensity sheath to fasten it. The minimum power scheme of the PMSM driver is shown in Figure 2. The six insulated gate bipolar transistors (IGBTs) VT1~VT6 are used as the power switches of the rectifier and inverter. The resistor R0 and relay KT compose the power soft-start module, while the resistors R1~R3 and capacitors C1~C3 are able to stabilize the DC-bus voltage Udc. The resistor RL is traded as the simulate load. In the three-phase interface of the motor electric pole, three current sensors U/V/W are connected to measure the stator current.

Based on this, the kinematic model for the surface-mounted PMSM (SPMSM) in the coordinate of three-phase static *a*/*b*/*c* can be described as follows:

(1) $$\left\{ \begin{array}{l} u_{s}=R_{s}i_{s}+L_{s}\frac{di_{s}}{dt}+\frac{d\psi_{s}}{dt},\\ \varphi_{s}=\varphi_{f}\left[\begin{array}{c} \sin(\omega_{r}t+\theta_{r})\\ \sin(\omega_{r}t+\theta_{r}+2\pi /3)\\ \sin(\omega_{r}t+\theta_{r}+4\pi /3) \end{array},\right] \end{array}\right.\;$$

where $u_{s}={\left[\begin{array}{ccc} u_{a} & u_{b} & u_{c} \end{array}\right]}^{T}$, $i_{s}={\left[\begin{array}{ccc} i_{a} & i_{b} & i_{c} \end{array}\right]}^{T}$ are stator three-phase voltage and current, respectively. $L_{s}$, $R_{s}$ are armature inductance and resistor. $\theta_{r}$, $\omega_{r}$ are angular position and speed.

**Figure 3:**
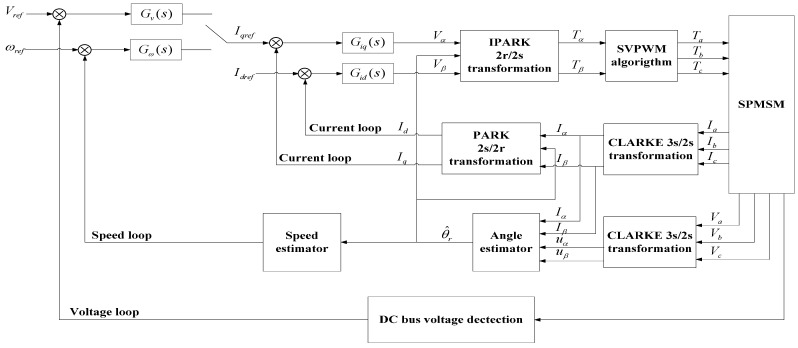
PID-based vector control block diagram. SPMSM: surface-mounted PMSM; SVPWM: space vector pulse width modulation.

Generally, the PID-based vector control strategy was adopted for the SPMSM system, including speed loop during charging (voltage loop during discharging), double current loop and position loop, as shown in Figure 3. $V_{ref}$, $\omega_{ref}$, $I_{qref}$ and $I_{dref}$ are voltage, speed and current reference inputs, respectively. $G_{v}(s)$, $G_{\omega }(s)$, $G_{iq}(s)$ and $G_{id}(s)$ are PID controllers.

In the vector control loop as shown in Figure 3, the coordinate transformations need to be disposed, including CLARKE transformation (three-phase *a*/*b*/*c* to two-phase static *α*/*β* coordinate, 3*s*/2*s*), PARK transformation (two-phase static *α*/*β* to two-phase rotating *d*/*q* coordinate, 2*s*/2*d*) with its inverse IPARK transformation. The kinematic model for the SPMSM in the coordinate of two-phase static *α/β* can be described as follows:

(2) $$\left\{ \begin{array}{l} u_{\alpha }=R_{s}i_{\alpha }+L_{s}\frac{di_{\alpha }}{dt}+e_{\alpha },\\ u_{\beta }=R_{s}i_{\beta }+L_{s}\frac{di_{\beta }}{dt}+e_{\beta },\\ e_{\alpha }=-\omega_{r}\varphi_{f}\sin\theta_{r},\\ e_{\beta }=\omega_{r}\varphi_{f}\cos\theta_{r}, \end{array}\right.\;$$

where $u_{\alpha }$, $u_{\beta }$, $i_{\alpha }$, $i_{\beta }$ are motor stator two-phase static voltage and current, respectively. $e_{\alpha }$, $e_{\beta }$ are motor back electromotive force in the *α/β* coordinate. $\varphi_{f}$ is the excitation flux linkage of the rotor permanent magnet.

According to the IPARK transformation, the kinematic model in the coordinate of two-phase rotating *d/q* can be formulated as:

(3) $$\left\{ \begin{array}{l} u_{d}=R_{s}i_{d}+L_{s}\frac{di_{d}}{dt}-\omega_{r}L_{s}i_{q},\\ u_{q}=R_{s}i_{q}+L_{s}\frac{di_{q}}{dt}+\omega_{r}L_{s}i_{d}, \end{array}\right.\;$$

where $u_{d}$, $u_{q}$, $i_{d}$, $i_{q}$ are motor stator two-phase rotating voltage and current, respectively. In this formulation, the variation of the permanent magnet can be ignored.

It is known that the rotor angular position $\theta_{r}$ is the crucial parameter in the coordinates transformation. Usually, the position sensor resolver or encoder is applied to obtain the angular position and speed. However, for the MSFESS, the rotor is suspended in the geometric center and hence there is a certain large distance between the motor stator and rotor. Accordingly, there are nearly no position sensors to satisfy this distance circumstance. In addition, the special sensors are usually expensive, which cannot be tolerated due to economic consideration. Therefore, the sensorless vector control becomes more and more important in practical engineering fields. In this paper, we focus on the real-time position estimation and compensation for the SPMSM based on the two-step extended slide-mode observer.

## 3. Basic Idea of This Work

The basic idea of this work is to estimate and compensate the angular position for the SPMSM by using the sensorless control strategy. Generally, the sensorless estimation approaches can be summarized as back electromotive force method, high-frequency signal injection, observers, etc. Among these methods, it has been shown that the observer-based sensorless estimation (SLE) is proved to be one of the most effective methods. For this purpose, the scheme of the traditional extended sliding-mode observer is first given as follows.

According to the relationships in Equation (2), the angular position and speed can be formulated as:

(4) $$\left\{ \begin{array}{l} \theta_{r}=-arc\tan\frac{e_{\alpha }}{e_{\beta }}\\ \omega_{r}=d\theta_{r}/dt, \end{array}\right.,\;$$

where it can be seen that the value of the position signal $\theta_{r}$ depends on the back electromotive forces $e_{\alpha }$ and $e_{\beta }$ in the *α/β* coordinate.

On the other hand, the PMSM system of the MSFESS is subjected to multi-source disturbances such as mass imbalance torque, magnetic bias force, gyroscope effect, etc. The disturbance torques have been introduced in [24] and are omitted here. The feedback current signals contain noise, which can also be disposed as disturbances. To achieve satisfactory control performance, the SLE system should be sensitive to position signal and simultaneously be robust to the unknown disturbances. 

Set the state variable as $x={\left[\begin{array}{cccc} i_{\alpha }, & i_{\beta }, & e_{\alpha }, & e_{\beta } \end{array}\right]}^{T}$, control input as $u={\left[\begin{array}{cc} u_{\alpha }, & u_{\beta } \end{array}\right]}^{T}$, and two-phase static current after CLARKE transformation as measured output $y={\left[\begin{array}{cc} i_{\alpha }, & i_{\beta } \end{array}\right]}^{T}$. For the PMSM, the mentioned disturbance torques act directly on the control forward loop, which can be equivalent to a disturbance voltage in the control input. Therefore, the unknown disturbance is composed of the disturbance voltage $v_{d}$ and measurement noise $n_{d}$, i.e., $d={\left[\begin{array}{cc} v_{d}, & n_{d} \end{array}\right]}^{T}$. In the case of unknown disturbance, the following state-space model is obtained based on the kinematic model in the *α/β* coordinate:

(5) $$\left\{ \begin{array}{l} \dot{x}=Ax+Bu+B_{d}d,\\ y=Cx+D_{d}d, \end{array}\right.\;$$

where

$$A=\left[\begin{array}{cccc} -\frac{R_{s}}{L_{s}} & 0 & -\frac{1}{L_{s}} & 0\\ 0 & -\frac{R_{s}}{L_{s}} & 0 & -\frac{1}{L_{s}}\\ 0 & 0 & 0 & -\omega_{r}\\ 0 & 0 & \omega_{r} & 0 \end{array}\right],B=\left[\begin{array}{c} \frac{1}{L_{s}}\\ \begin{array}{c} \frac{1}{L_{s}}\\ 0\\ 0 \end{array} \end{array}\right],B_{d}=\left[\begin{array}{cc} \frac{1}{L_{s}} & 0\\ \frac{1}{L_{s}} & 0\\ 0 & 0\\ 0 & 0 \end{array}\right],C=\left[\begin{array}{cccc} 1 & 0 & 0 & 0\\ 0 & 1 & 0 & 0 \end{array}\right],D_{d}=\left[\begin{array}{cc} 0 & 0.1\\ 0 & 0.1 \end{array}\right],\;$$

with the variance of the noise set to 0.1 according to the circuit condition. It is easy to know the plant is completely observable and hence the observer is excitable.

For the purpose of SLE, the following extended slide-mode observer is considered [25,26,27]:

(6) $$\left\{ \begin{array}{l} \dot{\hat{x}}=\hat{A}\hat{x}+B(u-u_{smo})\\ \hat{y}=C\hat{x}\\ u_{smo}=K_{slide}sat(y-\hat{y}) \end{array}\right.\;$$

where $\hat{x}$, $\hat{y}$ are the estimates of x and y, respectively. The observer matrix is $\hat{A}=\left[\begin{array}{cccc} -\frac{R_{s}}{L_{s}} & 0 & -\frac{1}{L_{s}} & 0\\ 0 & -\frac{R_{s}}{L_{s}} & 0 & -\frac{1}{L_{s}}\\ 0 & 0 & & -{\hat{\omega }}_{r}\\ 0 & 0 & {\hat{\omega }}_{r} & 0 \end{array}\right],$ with the estimated speed. The goal of the saturation control z is to drive current estimation error to zero. This is achieved by proper selection of the slide-mode gain $K_{slide}$ and correct formation of the estimated back emf.

Provided that the evaluation function is $z={\left[\begin{array}{cc} e_{e_{\alpha }}, & e_{e_{\beta }} \end{array}\right]}^{T}$ with the estimated error $e_{e_{\alpha }}=e_{\alpha }-{\hat{e}}_{\alpha }$, $e_{e_{\beta }}=e_{\beta }-{\hat{e}}_{\beta }$. Then we have

(7) $$z(s)=G_{zu}(s)u(s)+G_{zd}(s)d(s).\;$$

It is expected that the transfer matrix gain from the disturbance d(s) to evaluation function z(s) is minimal. It is the so-called *H_∞_* optimization control or sub-optimization control, i.e.,

(8) $${\left\|G_{zd}(s)\right\|}_{\infty }\to \min,\;\mathrm{or}\;{\left\|G_{zd}(s)\right\|}_{\infty }<\gamma .\;$$

Due to this, the impact of the unknown disturbance can be rejected within an adaptable range, even with the worst disturbance circumstance. In this paper, we propose an optimal SLE method based on the extended sliding-mode observer in order to accomplish SLE in the whole speed range.

**Remark** **1.** *It is seen that the α/β current mode has two basic states i_α_ and i_β_ as described in (2). To improve the state estimation accuracy, the back electromotive forces e_α_ and e_β_ can be extended new states as shown in (5). Therefore, it is usual to call this sliding mode observer of (6) as an extended observer and some references like [27] have proposed this observer.*

## 4. Design of Sensorless Estimation System

### 4.1. H_∞_ Optimization-Based Sensorless Estimation

In this section, we start with the design of SLE system by applying the traditional extended sliding-mode observer method for the SPMSM.

By defining $e=x-\hat{x}={\left[\begin{array}{cccc} e_{x_{\alpha }} & e_{x_{\beta }} & e_{e_{\alpha }} & e_{e_{\beta }} \end{array}\right]}^{T}$, it follows from (5)–(7) that:

(9) $$\left\{ \begin{array}{l} \dot{e}=\hat{A}e-Bu_{smo}+B_{d}d,\\ z=C_{z}e,\\ u_{smo}=K_{slide}sat(y-\hat{y}), \end{array}\right.\;$$

where the evaluation matrix $C_{z}=\left[\begin{array}{cccc} 0 & 0 & 1 & 0\\ 0 & 0 & 0 & 1 \end{array}\right]$. The estimation error $e_{x_{\alpha }}=x_{\alpha }-{\hat{x}}_{\alpha }$, $e_{x_{\beta }}=x_{\beta }-{\hat{x}}_{\beta }$, $e_{e_{\alpha }}=e_{\alpha }-{\hat{e}}_{\alpha }$, $e_{e_{\beta }}=e_{\beta }-{\hat{e}}_{\beta }$.

In Equation (9), the saturation function is denoted as follows:

(10) $$sat(y-\hat{y})=\left\{ \begin{array}{ll} -{\tilde{y}}_{th}, & (y-\hat{y})<-{\tilde{y}}_{th},\\ (y-\hat{y}), & {\tilde{y}}_{th}<(y-\hat{y})<{\tilde{y}}_{th},\\ {\tilde{y}}_{th}, & (y-\hat{y})>{\tilde{y}}_{th}, \end{array}\right.\;$$

where ${\tilde{y}}_{th}=y-\hat{y}$ is a preset constant.

According to (9) and (10):

(11) $$u_{smo}=\left\{ \begin{array}{ll} -K_{slide}{\tilde{y}}_{th}, & (y-\hat{y})<-{\tilde{y}}_{th},\\ K_{slide}(Ce+D_{d}d), & {\tilde{y}}_{th}<(y-\hat{y})<{\tilde{y}}_{th},\\ K_{slide}{\tilde{y}}_{th}, & (y-\hat{y})>{\tilde{y}}_{th}, \end{array}\right.\;$$

Then, the error equation can be rewritten as:

(12) $$\begin{array}{ll} (y-\hat{y})<-{\tilde{y}}_{th}, & \left\{ \begin{array}{l} \dot{e}=\hat{A}e+BK_{slide}{\tilde{y}}_{th}+B_{d}d,\\ z=C_{z}e, \end{array}\right.\\ \left|y-\hat{y}\right|<{\tilde{y}}_{th}, & \left\{ \begin{array}{l} \dot{e}=(\hat{A}-BK_{slide}C)e+(B_{d}-BK_{slide}D_{d})d,\\ z=C_{z}e, \end{array}\right.\\ (y-\hat{y})>{\tilde{y}}_{th}, & \left\{ \begin{array}{l} \dot{e}=\hat{A}e-BK_{slide}{\tilde{y}}_{th}+B_{d}d,\\ z=C_{z}e, \end{array}\right. \end{array}\;$$

From Equation (12), it can be seen that the observer stability depends on the system matrix $\hat{A}$ and $(\hat{A}-BK_{slide}C)$. This is the typical field of the modern control theory. In this paper, we focus on the transfer matrix from unknown disturbance d to evaluation function z is minimal, i.e., *H_∞_* optimization control. The case in $\left|y-\hat{y}\right|<{\tilde{y}}_{th}$ is considered with another special cases.

According to the traditional extended slide-mode observer in (6), the state estimates $\hat{x}=\left[\begin{array}{cccc} {\hat{i}}_{\alpha } & {\hat{i}}_{\beta } & {\hat{e}}_{\alpha } & {\hat{e}}_{\beta } \end{array}\right]$ can be obtained. Owing to the measurement of current $i_{\alpha }$ and $i_{\beta }$, the values of the errors $e_{x_{\alpha }}$ and $e_{x_{\beta }}$ can also be determined with $e_{x_{\alpha }}=i_{\alpha }-{\hat{i}}_{\alpha }$, $e_{x_{\beta }}=i_{\beta }-{\hat{i}}_{\beta }$.

Then, considering the errors $e_{x_{\alpha }}$ and $e_{x_{\beta }}$ as the measured output $y_{e}=\left[\begin{array}{cc} e_{x_{\alpha }} & e_{x_{\beta }} \end{array}\right]$, the error equation in (12) can be rewritten as

(13) $$\left|y-\hat{y}\right|<{\tilde{y}}_{th},\;\left\{ \begin{array}{l} \dot{e}=(\hat{A}-BK_{slide}C)e+(B_{d}-BK_{slide}D_{d})d,\\ y_{e}=C_{e}e,\\ z=C_{z}e, \end{array}\right.\;$$

where $C_{e}=\left[\begin{array}{cccc} 1 & 0 & 0 & 0\\ 0 & 1 & 0 & 0 \end{array}\right]$.

Generally, the sliding mode observer gain $K_{slide}$ is designed as follows [17,18,19]:

(14) $$K_{slide}>\max(\left|e_{\alpha }\right|,\left|e_{\beta }\right|),\;$$

where the sliding mode plane exists only if the gain $K_{slide}$ is larger than the back electromotive force in the *α/β* coordinate.

It is obvious that the formulation in (14) just provides the upper bound of the observer gain. The detailed value of the gain is not determined so that the states’ estimation accuracy cannot be guaranteed. In this way, the value of the gain can be approximately confirmed based on the formulation in (14).

Therefore, the error estimation equation can be further formulated as:

(15) $$\;\left\{ \begin{array}{l} \dot{\hat{e}}=A_{e}\hat{e}+L_{e}(y_{e}-{\hat{y}}_{e}),\\ {\hat{y}}_{e}=C_{e}\hat{e},\\ \hat{z}=C_{z}\hat{e}, \end{array}\right.\;$$

where the matrices $A_{e}=\hat{A}-BK_{slide}C$, $B_{e}=B_{d}-BK_{slide}D_{d}$ are constant matrices.

Combining Equations (13) and (15), it is obtained that:

(16) $$\;\left\{ \begin{array}{l} \dot{\tilde{e}}=(A_{e}-L_{e}C_{e})\tilde{e}+B_{d}d,\\ \tilde{z}=C_{z}\tilde{e}, \end{array}\right.\;$$

where the evaluation output error $\tilde{z}=z-\hat{z}$.

Based on this, the evaluation output is further expressed as:

(17) $$\tilde{z}(s)=G_{zd}(s)d(s),\;$$

where the transfer function matrix is:

$$G_{zd}(s)=C_{z}{\left(sI-A_{e}+L_{e}C_{e}\right)}^{-1}B_{d}.\;$$

**Lemma** **1.** *Considering the following linear system [23]*

$$\begin{array}{l} \dot{x}(t)=Ax(t)+Bw(t),\\ z(t)=Cx(t)+Dw(t), \end{array}\;$$

Given a positive coefficient γ>0, if there exists a positive matrix P>0 with the following linear matrix inequality satisfied:

$$\left[\begin{array}{ccc} A^{T}P+PA & PB & C^{T}\\ B^{T}P & -\gamma I & D^{T}\\ C & D & -\gamma I \end{array}\right]<0\;$$

Then, the original linear system is robustly stable and the following *H_∞_*-norm is met:

$$\left\|G_{zw}(s)\right\|<\gamma ,\;\forall w.\;$$

By using Lemma1, the error equation in (16) can be transferred as:

(18) $$\begin{array}{l} \gamma \to \min,\;s.t.\left[\begin{array}{ccc} {\left(A_{e}-L_{e}C_{e}\right)}^{T}P+P(A_{e}-L_{e}C_{e}) & PB_{d} & C_{z}^{T}\\ B_{d}^{T}P & -\gamma I & 0\\ C_{z} & 0 & -\gamma I \end{array}\right]<0,\\ P>0 \end{array}\;$$

The formulation in (18) can be considered as a traditional *H_∞_* optimization problem which is able to be disposed by the linear matrix inequality and objective function. Here, the detailed process is omitted.

**Remark** **2.** *It is certain that the H_∞_-optimization control method has been widely used in reality since the worst disturbance case has been considered. The influence of the disturbance on the estimation performance can be reduced effectively by using the H_∞_-optimization technique. But this approach has a little conservativenss, and is especially subject to a large estimation error. Due to this, this paper adapts a traditional observer as the first step to obtain the basic states estimations, and the second one is further designed to compensate the errors based on H_∞_ optimization control*.

### 4.2. Back Electromotive Force Error Compensation

After designing the observer gain $L_{e}$ by using the linear matrix inequality, the error $e={\left[\begin{array}{cccc} e_{x_{\alpha }} & e_{x_{\beta }} & e_{e_{\alpha }} & e_{e_{\beta }} \end{array}\right]}^{T}$ can be obtained. Then, putting the estimation error into the observer (13), we get

(19) $${\hat{e}}_{\alpha 1}={\hat{e}}_{\alpha }+e_{e_{\alpha }},\;{\hat{e}}_{\beta 1}={\hat{e}}_{\beta }+e_{e_{\beta }},\;$$

where ${\hat{e}}_{\alpha 1}$, ${\hat{e}}_{\beta 1}$ are estimates of back electromotive forces after compensation.

According to the relationship in (4), the estimates of the angular position and speed can be configured as:

(20) $$\left\{ \begin{array}{l} {\hat{\theta }}_{r}=-arc\tan\frac{{\hat{e}}_{\alpha 1}}{{\hat{e}}_{\beta 1}},\\ {\hat{\omega }}_{r}=\frac{d{\hat{\theta }}_{r}}{dt}. \end{array}\right.\;$$

Due to the measurement noise in the current $i_{\alpha }$ and $i_{\beta }$, it is inevitable that the estimates of the back electromotive forces ${\hat{e}}_{\alpha }$ and ${\hat{e}}_{\beta }$ contain much high-frequency disturbances. For this problem, the traditional method is to design a low-pass filter to reject the noise.

(21) $${\hat{\theta }}_{r}=-arc\tan\frac{{\hat{e}}_{\alpha 1}}{{\hat{e}}_{\beta 1}}*f_{filter}(s).\;$$

Usually, the low-pass filter is designed as a one-order filter as follows:

(22) $$f_{filter}(s)=\frac{\omega_{c}}{s+\omega_{c}},\;$$

where $\omega_{c}$ is the cut-off frequency.

It is known that there is some phase delay in the low-pass filter, resulting in a postponed angle estimation error. In some references, the phase compensation method has been used to make up the angle error as follows:

(23) $$\Delta \theta_{r}=\arctan\left(\frac{{\hat{\omega }}_{r}}{\omega_{c}}\right).\;$$

Based on this, the angular position after compensation is determined as:

(24) $${\hat{\theta }}_{r}=-\arctan\left(\frac{{\hat{e}}_{\alpha 1}}{{\hat{e}}_{\beta 1}}\right)+\arctan(\frac{{\hat{\omega }}_{r}}{\omega_{c}}).\;$$

**Figure 4:**
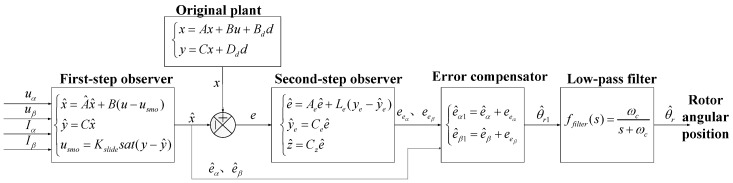
Two-step angular position estimation and compensation.

In this way, the two-step angle error estimation and compensation strategy can be accomplished. The control scheme block is shown in Figure 4. The overall angular position estimation and compensation algorithm can be summarized in Algorithm 1.
**Algorithm 1.** Given the original system (5) and a sampling period T_s_, set computational number k=0 and initial time $t_{1}=t_{2}=0$. The observer gain matrix $K_{slide}$ and L have been calculated based on formulation (14) and Lemma 1.Step 1. Sample the Hall sensor to refresh the measurements $i_{a}、i_{b}、i_{c}、V_{a}、V_{b}、V_{c}$. Step 2. Substitute $i_{a}、i_{b}、i_{c}、V_{a}、V_{b}、V_{c}$ into (2) to calculate the parameters $i_{\alpha }、i_{\beta }、V_{\alpha }、V_{\beta }$ in the *α/β* coordinate. Step 3. Put $i_{\alpha }、i_{\beta }、V_{\alpha }、V_{\beta }$ into (6) to calculate the estimation parameters ${\hat{i}}_{\alpha }、{\hat{i}}_{\beta }、{\hat{e}}_{\alpha }、{\hat{e}}_{\beta }$. Step 4. Substitute ${\hat{i}}_{\alpha }、{\hat{i}}_{\beta }、{\hat{e}}_{\alpha }、{\hat{e}}_{\beta }$ into (15) to calculate the error estimation parameters ${\hat{e}}_{i_{\alpha }}、{\hat{e}}_{i_{\beta }}、{\hat{e}}_{e_{\alpha }}、{\hat{e}}_{e_{\beta }}$. Step 5. Combine ${\hat{e}}_{e_{\alpha }}、{\hat{e}}_{e_{\beta }}$ into (19) to obtain the error compensation parameters ${\hat{e}}_{\alpha 1}、{\hat{e}}_{\beta 1}$. Step 6. Design the low-pass filter in (22) to compensate the error compensation parameters ${\hat{e}}_{\alpha 1}、{\hat{e}}_{\beta 1}$ to accomplish the position estimation and compensation according to (24).

**Remark** **3.** *For the traditional application, the first-step observer is suitable for a medium speed or frequency circumstance. In this case, it is not essential for the second-step observer combination. The existence of the proposed observer is always true since the original system is robustly stable. For the MSFESS, the design of the second-step observer can further improve the angle estimation accuracy*.

## 5. Experimental Results

### 5.1. Experimental Setup

**Figure 5:**
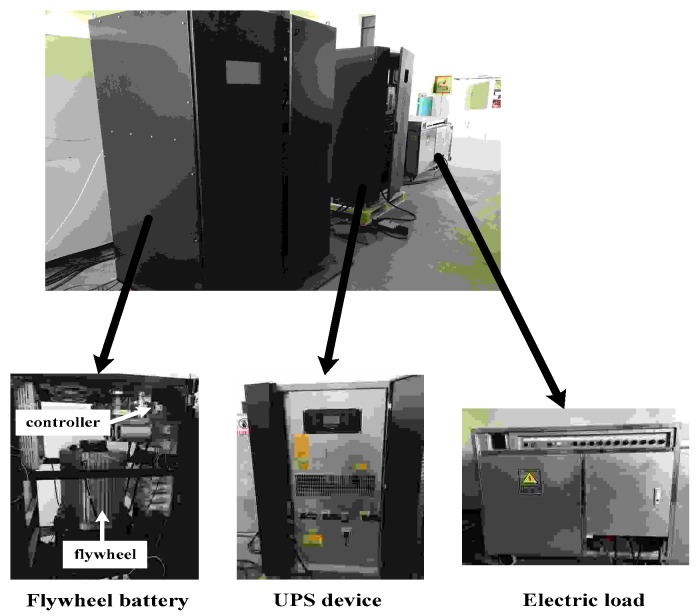
Experimental setup. UPS: unterruptible power supply.

The experimental setup is shown in Figure 5. The MSFESS consists of an aluminium outermost shell, inside PMSM and 5-freedom AMB, charging-discharging power unit and controller, master control screen and the outside black cabinet. The input power is 540 VDC voltage with the bus link, while the assistant 3-phase 380 VAC power is provided for AMB, vacuum and fan systems. The protection earth is configured near to the front door for prevention of the electric leakage. All of the required parameters are shown in Table 1.

Accordingly, the single gimbal system (4) can be governed with the following matrices:

$$A=\left[\begin{array}{cccc} -333.3 & 0 & -3333.3 & 0\\ 0 & -333.3 & 0 & -3333.3\\ 0 & 0 & 0 & -2198\\ 0 & 0 & 2198 & 0 \end{array}\right],B=\left[\begin{array}{c} 3333.3\\ \begin{array}{c} 3333.3\\ 0\\ 0 \end{array} \end{array}\right],B_{d}=\left[\begin{array}{cc} 3333.3 & 0\\ 3333.3 & 0\\ 0 & 0\\ 0 & 0 \end{array}\right],C=\left[\begin{array}{cccc} 1 & 0 & 0 & 0\\ 0 & 1 & 0 & 0 \end{array}\right],D_{d}=\left[\begin{array}{c} 0.1\\ 0.1 \end{array}\right].\;$$

Based on the rated voltage of the MSFESS in Table 1, the back electromotive forces can be near to $600/\sqrt{3}=346.4(VAC)$, i.e., $e_{a},\;e_{b},\;e_{c}\leq 346.4(VAC)$. After CLARKE transformation 3*s*/2*s*, the back electromotive forces in coordinate can be estimated as $\max(e_{\alpha },\;e_{\beta })\leq \max(e_{a},\;e_{b},\;e_{c})$. Therefore, the first-step observer gain $K_{slide}$ can be designed as $K_{slide}=346.4$.

After that, by using the LMI box tool in MATLAB software, the solution of the inequality in Lemma 1 can be obtained exactly. Based on that, the positive coefficient and matrix can be calculated as:

$$\gamma =0.01,\;P=\left[\begin{array}{cccc} -333.3 & 0 & -3333.3 & 0\\ 0 & -333.3 & 0 & -3333.3\\ 0 & 0 & 0 & -2198\\ 0 & 0 & 2198 & 0 \end{array}\right].$$

### 5.2. Angular Position Estimation Results

**Figure 6:**
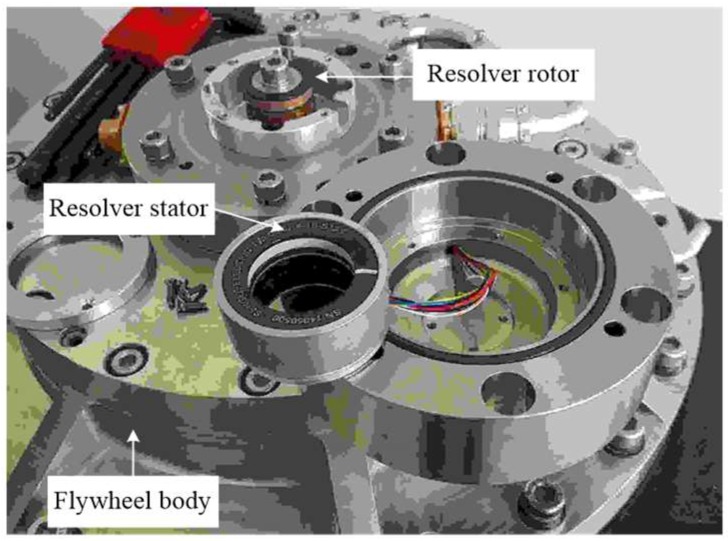
Resolver in the MSFESS.

For the MSFESS, the flywheel can be accelerated to a high speed when the magnetically suspended system can function properly since the flywheel axis will strike the bearing without suspension. However, when the motor is controlled in a low speed, it can also be safe even if the AMB system is powered off and in that case, the flywheel rotor will stay connected with the bearing body. For this working condition, a kind of position sensor can be installed at the terminal of the rotor axis and applied as the angle benchmark to evaluate the observer estimation result. Figure 6 shows the real picture of the resolver, consisting of a stator and rotor, while the rotor is fixed at the terminal of the axis and the stator is connected to the flywheel body. However, if the flywheel axis is suspended by the AMB system, the clearance between the stator and rotor will exceed the maximum tolerance value of the resolver, and in this case, the resolver cannot be used any more.

**Figure 7:**
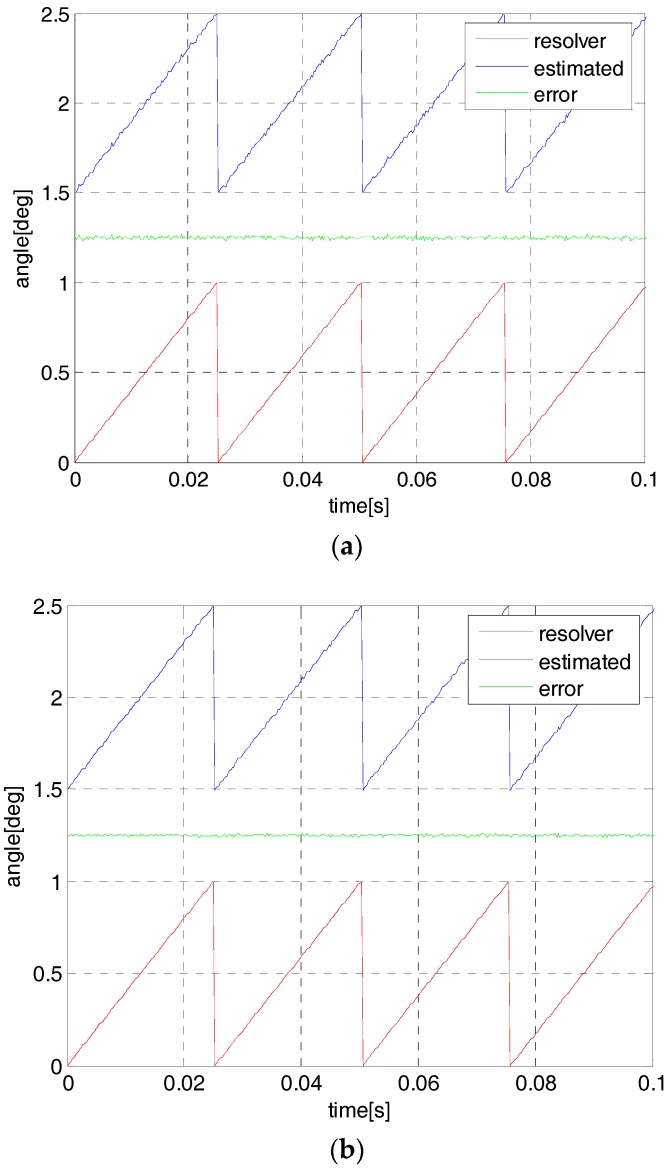
The simulated angle and its estimation at 1200 rpm by using one-step observer (**a**) and two-step observer (**b**).

**Figure 8:**
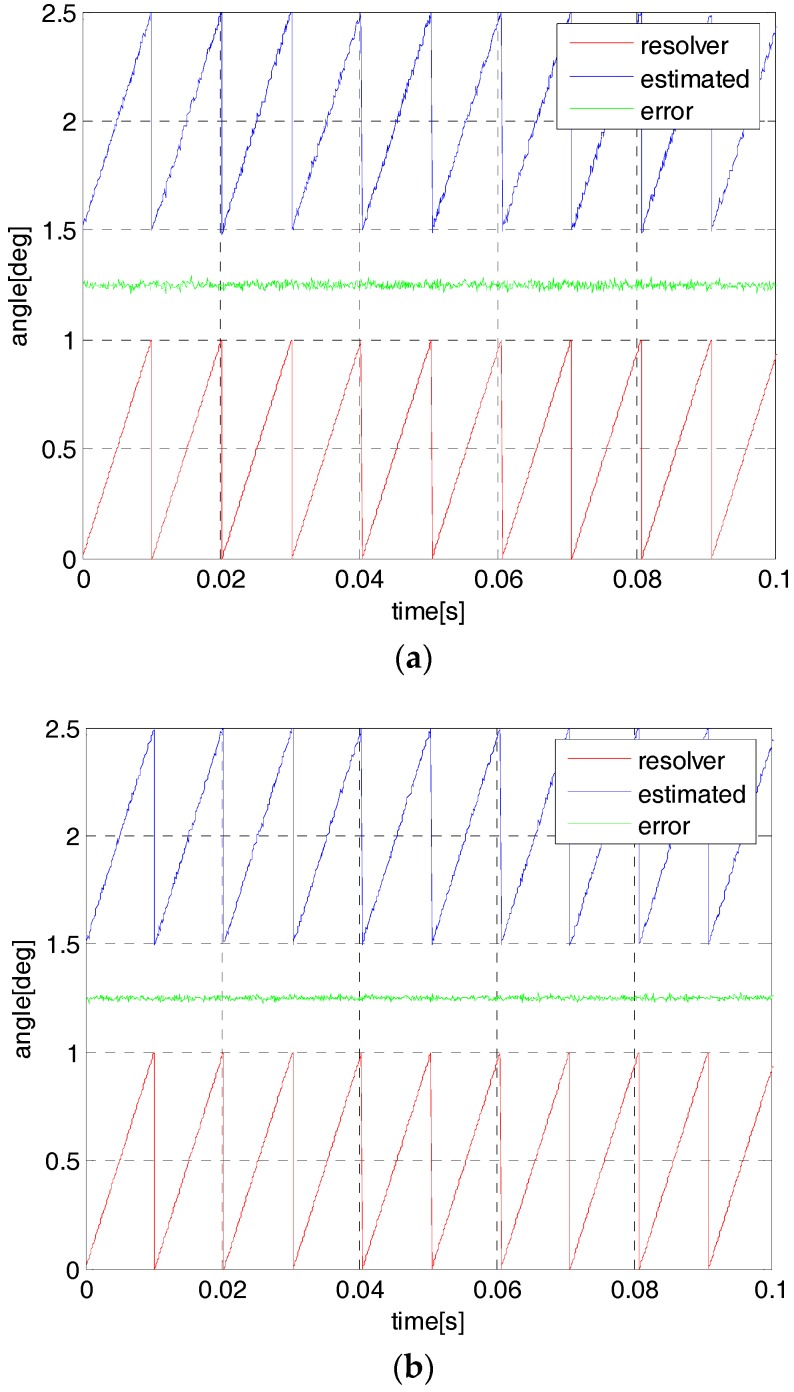
The simulated angle and its estimation at 3000 rpm by using one-step observer (**a**) and two-step observer (**b**).

For the sensorless control, the SPMSM will be started in an open-loop mode since there is no initial current in the motor stator and the observer has no angle estimation. In the open-loop start-up mode, a simulated angle function is generated by the program code and the motor will rotate gradually according to the frequency value of the angle function. The experiment is developed to compare the estimated performance by using one-step and the proposed two-step observer. Figure 7 and Figure 8 show the resolver angle and its estimation by the proposed observer. In this condition, the flywheel speed is set to 1200 rpm and 3000 rpm with the angle frequency 40 Hz and 100 Hz, respectively. Actually, the motor can be started in the position sensor mode but it is not practical for the sensorless control strategy. In these curves, the resolver angle is located in the field of 0~1, i.e., 0~360 deg (red line) and a constant of 1.5 has been added to the estimated angles for simplicity (blue line), while the green line is the estimated angle error.

It is seen that at both 1200 rpm and 3000 rpm, the estimated angular position can track the simulated and resolver angles. At a low speed, the simulated angle can be accurate with respect to the real resolver angle but it is not correct in a high-speed range. Additionally, it is not obvious in the estimation performance by using the two methods since the common sliding-mode observer is accurate enough for the low speed circumstance.

**Figure 9:**
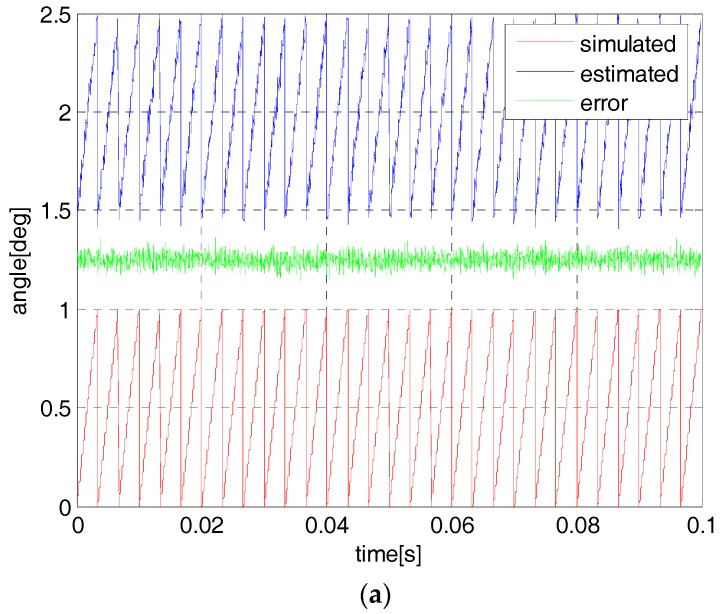

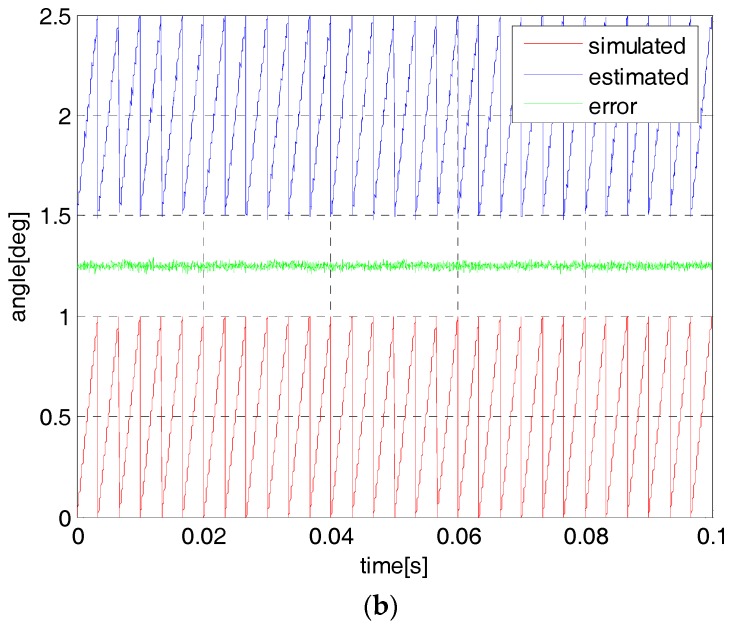
The simulated angle and its estimation at 9000 rpm by using one-step observer (**a**) and two-step observer (**b**).

In order to evaluate the estimation performance of the proposed observer, the resolver sensor is removed and the AMB system is powered for flywheel full-suspension. Due to this, the flywheel speed is set to 9000 rpm with the angle frequency 300 Hz. Also, the open-loop start-up method is used to achieve the typical V/F control algorithm. Figure 9 shows the estimation results by using the simulated and estimation strategy. It is demonstrated that the estimated value by using the one-step observer is not perfect with a lagging phase and fluctuant magnitude; there is much noise in the estimation results. By using the proposed two-step observer, the estimated angular position can also be appropriate for the simulated value, and the estimated results are smoother, which are effective for the control application.

### 5.3. Control Results

**Figure 10:**
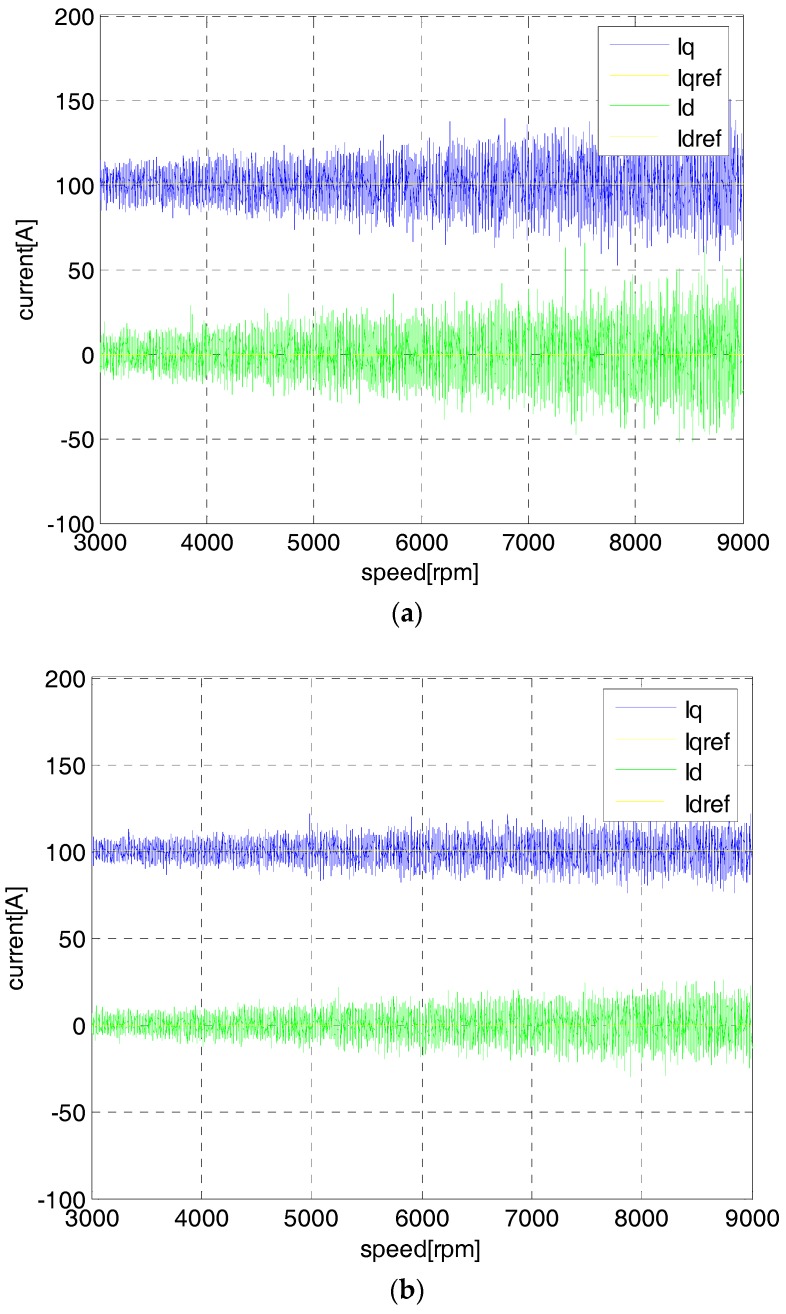
The close-loop control performance at 9000 rpm by using one-step observer (**a**) and two-step observer (**b**).

In order to test the control performance by using the sensorless control strategy, the estimated angular position is fed back to the control loop in Figure 3 to accomplish the flywheel acceleration process. The flywheel speed is set to the medium-speed 9000 rpm and the two steps observer are experimented. In the closed-loop control structure, the *I_q_* and *I_d_* currents are the main control objects. When the currents in the *d*/*q* coordinate are controlled perfectly, the control performance of the outer speed loop or voltage loop will be better. Therefore, the control results of the *I_q_* and *I_d_* current loops are able to indicate the control performance improvement by using the two steps for the sliding-mode observer. In the charging mode with the speed from 0 to 9000 rpm, Figure 10 shows the current measurements of *I_q_* and *I_d_*, while the yellow line is the reference *I_qref_* with the blue one *I_q_* the feedback value, and the green one is the *I_d_* feedback value with its reference *I_dref_* zero.

In the charging mode of the MSFESS, the constant torque strategy is used to accelerate the rotor speed. In this case, the effective charging current remains constant about *I_qref_* = 100 A (as shown in Figure 10) so that the ideal *I_q_* feedback value could be used to track the reference value *I_qref_*. The *I_qref_* = 0 strategy is adapted to minimize the invalid current. It can be seen from Figure 10 that both the *I_q_* and *I_d_* current measurements have larger fluctuations, while the maximum of the *I_q_* feedback value can reach 150 A with 50% overshot and the maximum overshot of the *I_d_* feedback value can also be worse than 55%. These control conditions cannot be applied in the flywheel charging-discharging process. However, by using the proposed two-step observer, both the fluctuations of the *I_q_* and *I_d_* current measurements can be largely reduced, and the maximum of the *I_q_* feedback value can be less than 110 A with 10% overshot, while maximum overshot of the *I_d_* feedback value can also be better than 15%. These control conditions can satisfy the flywheel rapid charging-discharging requirements. Above all, the proposed sensorless control algorithm can improve the control performance of the MSFESS in the high-speed field through the two-step sliding-mode observer and *H_∞_* control method.

## 6. Conclusions

In this paper, the problem of angular position estimation in the sensorless control strategy has been studied for the MSFESS. Through analysis of the system modeling and principle description, it is obvious that the sensorless control has to be essential for the MSFESS based on the rotor angular position estimation strategy. Compared with other estimation methods, the sliding-mode observer algorithm has the advantages of robustness, reliability and simplicity. Based on this, a kind of two-step sliding-mode observer has been designed, including a traditional first-step sliding-mode and an extended second-step observer. In this scheme, the first step is used to guarantee the stability and convergence of the observer, while the second one can improve the estimation accuracy of the states. For the second-step observer design, the *H_∞_* optimization approach has been used based on the linear matrix inequality solving such that the influence of the unknown disturbances on the state estimation accuracy can be rejected within a low-level range. Finally, real experiments have been developed to demonstrate the effectiveness of the proposed method. It is shown that the angular position estimation accuracy can be further improved by using the two-step sliding-mode observer compared with the one step. Thus, the control results in both charging and discharging processes can also satisfy the desired performance.

## Figures and Tables

**Table 1:** MSFESS parameters.

Parameter	Index	Symbol
Electric capacity	3 kWh	*E_N_*
Rated speed	10,500 rpm	*n_N_*
Rated power	250 kVA	*P_N_*
Rated voltage	600 V	*V_N_*
Rated current	255 A	*I_N_*
Stator resistance	0.1 Ω	*Rs*
Stator inductance	300 μH	*Ls*
Pole-pairs	2	*p*
Rotor moment of inertia	16.2 kg·m^2^	*J_N_*
Bearing type	AMB	-
Vacuum value	<10 Pa	-
